# Vitrectomy for macular retinoschisis associated with peripapillary intrachoroidal cavitations in a moderately myopic eye

**DOI:** 10.1186/s40942-022-00409-w

**Published:** 2022-09-05

**Authors:** Shuichiro Aoki, Hiroko Imaizumi

**Affiliations:** 1 Department of Ophthalmology, Sapporo City General Hospital, 13-1 Kita 11 West, Chuo-ku, Sapporo, 060-8604 Japan; 2 Department of Ophthalmology, University of Tokyo Graduate School of Medicine, 7-3-1 Hongo, Bunkyo-ku, Tokyo, 113-8655 Japan

**Keywords:** Foveal detachment, Internal limiting membrane peeling, Macular retinoschisis, Peripapillary intrachoroidal cavitation

## Abstract

**Background:**

Peripapillary intrachoroidal cavitation (PICC), a cavernous change in the peripapillary choroid, may present with macular lesions. Here, we present a rare case of PICC with outer lamellar macular hole and macular retinoschisis.

**Case presentation:**

A 69-year-old man presented with metamorphopsia in the left eye. Fundus examination revealed macular retinoschisis and PICCs. Pars plana vitrectomy with fovea sparing internal limiting membrane peeling (FSIP) was performed. Three weeks postoperatively, the patient developed rhegmatogenous retinal detachment and underwent a second vitrectomy. The macular retinoschisis resolved without macular hole formation and the patient’s visual acuity improved. There were no recurrent macular lesions over the three years of postoperative follow-up. Postoperative spectral-domain optical coherence tomography scan revealed a communication between the PICC and the vitreous cavity.

**Conclusions:**

This case shed light on understanding development of PICC in non-highly myopic eyes and a rare complication of macular retinoschisis. Vitrectomy with FSIP may effectively resolve macular retinoschisis.

## Introduction

Peripapillary intrachoroidal cavitation (PICC) is a cavernous change in the peripapillary choroid [1, 2] and is mainly observed in highly myopic eyes [3]. Eyes with PICC may present with macular retinoschisis or macular detachment [4]. Herein, we present an unusual case of macular retinoschisis in a moderately myopic eye with bilateral PICC. The case is unique in that an opening of the PICC into the vitreous cavity appeared postoperatively, years after the retinoschisis had improved.

## Case report

**Fig. 1:**
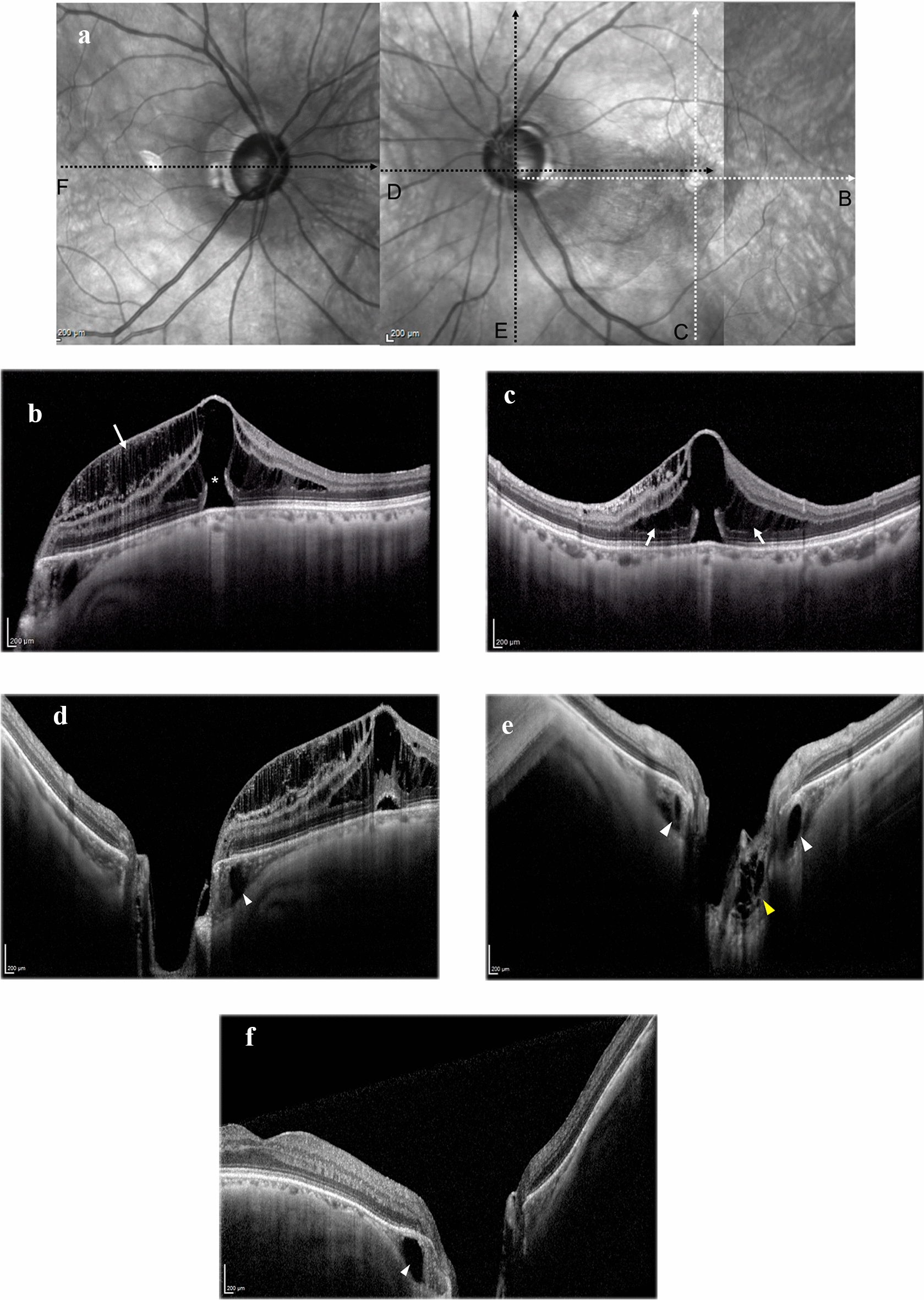
Baseline optical coherence tomography (OCT) imaging. **A** Near-infrared reflectance imaging of both eyes at initial presentation. **B**, **C** Spectral-domain (SD-OCT) line scans (indicated by white dotted arrows in **A**) through the fovea showing macular retinoschisis (white arrows) and the outer lamella hole (asterisk). SD-OCT via the optic nerve head in the left (**D**, **E**) and the right (**F**) eyes (indicated by black dotted arrows in **A**). The peripapillary sclera is positioned posteriorly relative to the retinal pigment epithelium, thickened peripapillary choroid, and PICCs (white arrowheads). A honeycomb-like structure was observed in the prelaminar region of the left eye (**E**, yellow arrowhead)

A 69-year-old man was referred to our hospital with complaints of central vision loss and metamorphopsia in the left eye for three weeks. Several years earlier, the patient underwent vitrectomy and lens reconstruction surgery in the right eye for retinal detachment. The assessment at admission to our department revealed a best-corrected visual acuity of 1.0, with a refraction of − 1.0 diopter sphere with − 1.0 diopter cylinder at 90° in the right eye (OD) and 0.2 with a refraction of − 2.5 diopter cylinder at 80° in the left eye (OS). The axial length was 25.90 mm OD and 25.00 mm OS. The intraocular pressure was 18 mmHg OD and 20 mmHg OS. Slit-lamp examination revealed no abnormality, except for the presence of an intraocular lens in the right eye and mild cataract in the left eye. Dilated fundus examination revealed macular retinoschisis and a suspected macular hole with posterior vitreous detachment in the left eye. The optic nerve head in both eyes exhibited moderate rim thinning with a nerve fiber layer defect at the inferotemporal margin. Retinal breaks with chorioretinal scars were observed on the peripheral fundus of the right eye. Spectral-domain optical coherence tomography (SD- OCT) showed an outer lamellar macular hole with foveal retinal detachment (Fig. 1A–C). Retinoschisis was observed at the outer plexiform layer around the fovea and the inner retinal layer between the optic disc and fovea. OCT line scans via the optic disc in both eyes (Fig. 1D, E) revealed deep cupping of the optic nerve head and posterior displacement of the peripapillary sclera relative to the retinal pigment epithelium. PICCs were observed inside the thickened peripapillary choroid (Fig. 1D–F) and a honeycomb-like structure was observed in the prelaminar region of the left eye (Fig. 1E). At the initial evaluation, neither optic disc pits nor communication among the vitreous cavity, PICC, and intraretinal space were observed in both eyes.

**Fig. 2:**
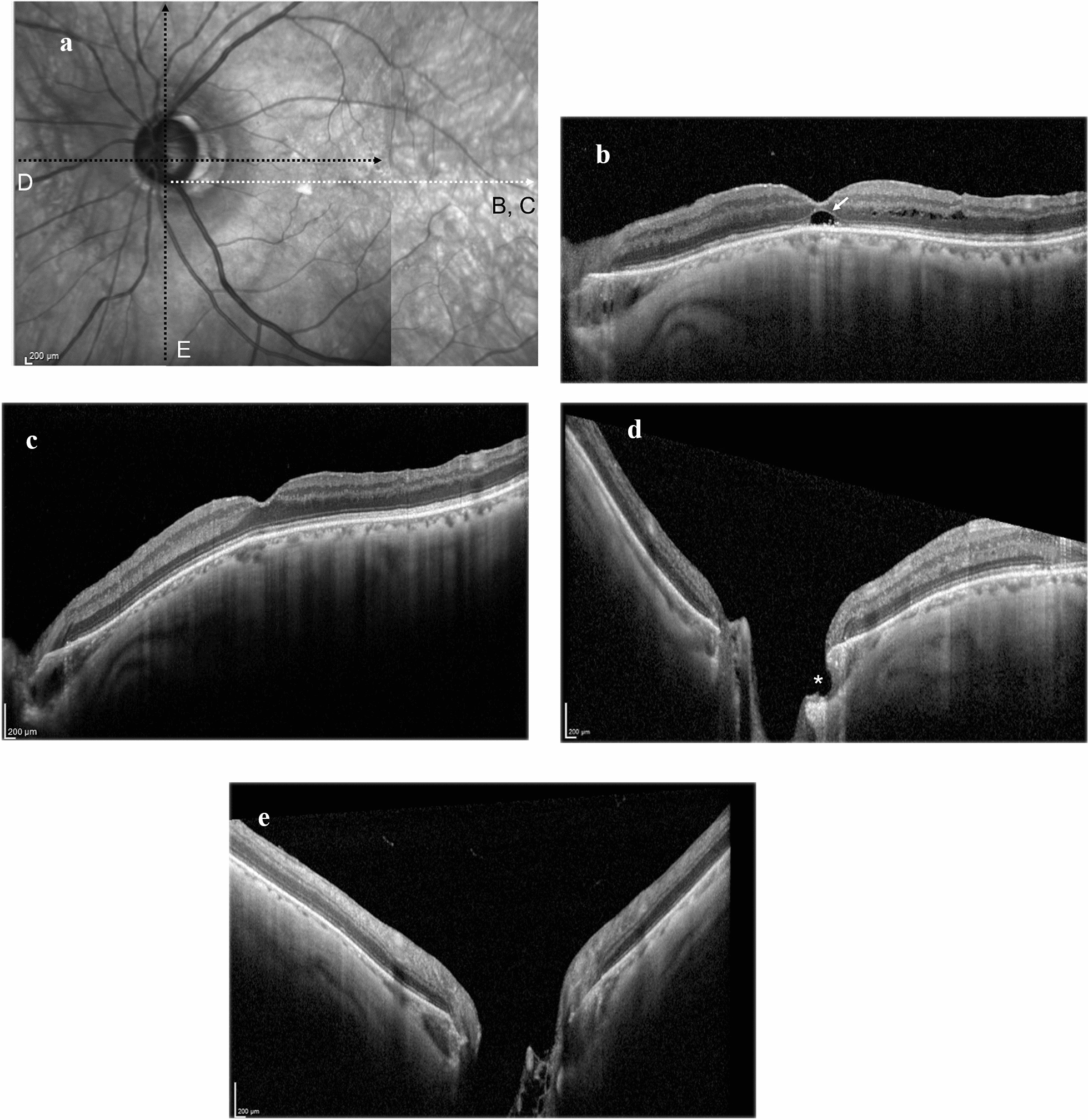
Near-infrared reflectance imaging (**A**) and SD-OCT line scans through the fovea (**B** and **C** indicated by a white dotted arrow) and the optic nerve head (**D** and **E** indicated by black dotted arrows) of the left eye. **B** Macular retinoschisis improved and foveal detachment remained (white arrow) 2 weeks after the first surgery. **C**–**E** Two years postoperatively, macular retinoschisis resolved and an opening (asterisk in **D**) of the temporal PICC into the vitreous cavity was observed

Based on these observations, the macular lesion was considered to have been caused by an anomaly of the optic nerve head. Our service indicated pars plana vitrectomy with internal limiting membrane peeling and sulfur hexafluoride gas tamponade to address the pathology. During surgery, complete posterior vitreous detachment around the optic disc was observed, consistent with the slit-lamp examination findings at presentation. The fovea-sparing internal limiting membrane peeling (FSIP) technique and subsequent fluid-gas exchange using a 12% sulfur hexafluoride gas tamponade were administered. The patient remained in the prone position for 2 days postoperatively. Two weeks postoperatively, the macular retinoschisis had improved, but the foveal detachment remained (Fig. 2B). In the third postoperative week, the patient underwent another vitrectomy due to complications, namely, rhegmatogenous retinal detachment with macular detachment. Tamponade using 20% sulfur hexafluoride gas was performed. Postoperatively, no full-thickness macular hole formation was observed and the macular retinoschisis resolved (Fig. 2C). Corrected visual acuity in the left eye had improved to 0.6. During the three years of follow-up, no recurrent macular lesions were observed.

OCT scans of the optic disc of the left eye three years postoperatively demonstrated changes in the curvature of peripapillary retinal pigment epithelium and sclera compared to the baseline OCTs and a communication between the temporal PICC and vitreous cavity (Fig. 2D), which had not been observed during the initial examination.

The patient provided written informed consent for the publication of this case, including images.

## Discussion and conclusions

We encountered a rare case of macular retinoschisis with PICC that was successfully resolved with surgery. PICC is a cavernous lesion in the peripapillary choroid, first described by Freund et al. [1] as an elevated, orange-yellow lesion observed at the border of the myopic conus. On OCT, PICC presents as a hyporeflective space without alteration of the retinal pigment epithelium. Macular retinoschisis is a rare complication of PICC [4, 5]. In previous case reports of PICC-associated macular retinoschisis or macular detachment, the pathogenesis of macular abnormalities has been postulated to be fluid entry into the subretinal or intraretinal space through communication between the PICC and vitreous cavity [4, 5], or the traction of membrane tissue along that pathway [6]. In the present case, no communication was detected preoperatively between the vitreous cavity, PICC, and intraretinal or subretinal space. Nevertheless, potential communication may have led to the formation of the macular lesion.

Macular retinoschisis develops in various ocular disorders, including pathologic myopia and congenital or acquired optic disc anomalies such as optic disc pit [7, 8], coloboma, morning glory disc [9], and glaucoma [10]. The current case could classified as an optic disc anomaly because it presented with not only PICC, but also associated features, such as deep cupping of the optic nerve head and increased curvature of the peripapillary sclera and the latter progressed over three years. These features have been reported in eyes with high myopia by Spaide et al. [11], and the authors hypothesized that the PICC forms secondary to posterior scleral deformation. The present case is rare in that the anomalous configuration was observed in a pair of moderately myopic eyes.

Vitrectomy is an effective management method for macular retinoschisis associated with optic disc anomaly and PICC. It is postulated that releasing traction by the vitreous or epipapillary membrane on or around the optic nerve head prevents fluid from entering intraretinal or subretinal spaces [4, 6, 12–15]. However, the role of vitreous traction in the pathogenesis of macular retinoschisis remains controversial [16]. Some studies have reported that vitrectomy is effective for macular retinoschisis in which no obvious pit was detected [17] or for idiopathic macular retinoschisis [18]. Thus, the mechanism by which vitrectomy resolves macular lesions is not yet fully understood. In the present case, posterior vitreous detachment was observed preoperatively and there was no traction of membranous tissue on the optic nerve head. However, internal limiting membrane (ILM) peeling might have changed the traction on the peripapillary retina, thereby reducing fluid entry into the subretinal or intraretinal space through a potential communication, contributing to the improvement of the condition. ILM peeling may also facilitate the dispersion of subretinal and intraretinal fluids into the vitreous cavity. Some reports question the usefulness of gas tamponade and ILM peeling for macular retinoschisis associated with pit maculopathy [14, 19]. Vitrectomy alone might have improved the macular retinoschisis in the present case.

Macular holes are one of the most serious postoperative complications of vitrectomy with ILM peeling for myopic traction maculopathy [20, 21]. FSIP reduce the risk of postoperative full-thickness macular hole in vitreous surgery for vitreomacular traction syndrome and macular retinoschisis in highly myopic eyes [22–24]. In the present case, an outer lamellar macular hole was observed preoperatively, and the ILM was the only intact foveal tissue. The outer lamellar macular hole is characterized by the disruption of the external limiting membrane with a hole in the outer retina [25, 26]. It usually presents as a vitreomacular traction disorder, where foveal traction from a partially detached posterior hyaloid causes the elevation of the inner Müller cell layer of the foveola without its disruption; subsequent elevation of the inner layers of the foveal walls produces stretching of Müller cells, resulting in centrifugal traction on the outer nuclear layer and a hole in the outer retina [25–27]. However, some reports suggest that the outer lamellar hole following serous retinal detachment precedes full-thickness macular hole formation [28] and is also involved in the progression of myopic tractional maculopathy to a full-thickness macular hole [21, 29]. The outer lamellar hole observed in the present case was likely secondary to macular retinoschisis or serous retinal detachment. FSIP was performed because of the high risk of postoperative full-thickness macular hole formation [21]; after the initial surgery, the patient developed rhegmatogenous retinal detachment with macular detachment, but with no full-thickness macular hole. When performing ILM peeling for macular retinoschisis, FSIP should be considered, while considering the risk of complications, such as a full-thickness macular hole.

In conclusion, PICC with an anomalous configuration of the optic nerve head and peripapillary sclera in a non-highly myopic eye can cause macular retinoschisis. FSIP might have contributed to the resolution of macular retinoschisis while preventing complications.

## Data Availability

All data are available on reasonable request to the corresponding author.

## Abbreviations

FSIP: Fovea sparing internal limiting membrane peeling
ILM: Internal limiting membrane
OCT: Optical coherence tomography
PICC: Peripapillary intrachoroidal cavitation
